# Alpha synuclein overexpression can drive microbiome dysbiosis in mice

**DOI:** 10.1038/s41598-024-82139-7

**Published:** 2025-02-01

**Authors:** Timothy R. Sampson, Zachary D Wallen, Woong-Jai Won, David G. Standaert, Haydeh Payami, Ashley S. Harms

**Affiliations:** 1 Department of Neurology, University of Alabama at Birmingham, Birmingham, AL 35233 USA; 2 Aligning Science Across Parkinson’s (ASAP) Collaborative Research Network, Chevy Chase, MD 20815 USA; 3 Department of Cell Biology, Emory University School of Medicine, Atlanta, GA 30322 USA

**Keywords:** Metagenomics, Alpha-synuclein, Transgene, Parkinson’s disease, Gut microbiome, Age-related dysbiosis, Neuroscience, Microbiology, Bacteria

## Abstract

Growing evidence indicates that persons with Parkinson disease (PD), have a unique composition of indigenous gut microbes. Given the long prodromal or pre-diagnosed period, longitudinal studies of the human and rodent gut microbiome before symptomatic onset and for the duration of the disease are currently lacking. PD is partially characterized by the accumulation of the protein α-synuclein (α-syn) into insoluble aggregates, in both the central and enteric nervous systems. As such, several experimental rodent and non-human primate models of α-syn overexpression recapitulate some of the hallmark pathophysiologies of PD. These animal models provide an opportunity to assess how the gut microbiome changes with age under disease-relevant conditions. Here, we used a transgenic mouse strain, which overexpress wild-type human α-syn to test how the gut microbiome composition responds in this model of PD pathology during aging. Using shotgun metagenomics, we find significant, age and genotype-dependent bacterial taxa whose abundance becomes altered with age. We reveal that α-syn overexpression can drive alterations to the gut microbiome composition and suggest that it limits diversity through age. Taxa that were most affected by genotype-age interaction were *Lactobacillus* and *Bifidobacteria*. In a mouse model, we showed direct link between alpha synuclein geneotype (hallmark of PD), a dysbiotic and low-diversity gut microbiome, and dysbiotic levels of *Bifidobacteria* and *Lactobacillus* (most robust features of PD microbiome). Given emerging data on the potential contributions of the gut microbiome to PD pathologies, our data provide an experimental foundation to understand how the PD-associated microbiome may arise as a trigger or co-pathology to disease.

**Supplementary Information:**

The online version contains supplementary material available at 10.1038/s41598-024-82139-7.

## Introduction

Parkinson disease (PD) is a common, progressive, neurodegenerative movement disorder. Pathologically, PD is characterized by the loss of dopamine-producing neurons in the substantia nigra pars compacta (SNpc) and neuronal accumulation of pathological forms of the protein α-synuclein (α-syn) into insoluble aggregates, termed Lewy bodies. This accumulation of α-syn results in numerous cellular and immune dysfunctions and eventually neuronal cell death in the SNpc, as well as in other brain regions. While most cases of PD are idiopathic, genetic mutations or duplications in the genes encoding α-syn, *SNCA*, which increase expression or result in the disruption of the native structure of α-syn, lead to familial forms of PD^1^. Genome-wide association studies (GWAS) show strong associations between expression quantititave trait loci (eQTLs) in or near *SNCA* that affect the expression of α-syn and increased risk of idiopathic PD^2,3^. Various experimental rodent and non-human primate models of α-syn overexpression recapitulate some of these physiologic and pathologic characteristics^4,5^. Collectively, these data point to a central role for α-syn in the etiology of PD, but the mechanisms by which α-syn initiates or drives disease pathogenesis are unknown.

Gastrointestinal (GI) dysfunction is a prevalent prodromal symptom of PD observed either preceding and/or concurrent with diagnostic motor symptoms. Common symptoms such as gastroparesis and chronic constipation persist for the duration of the disease, which impacts patient quality of life. While α-syn aggregation in the basal ganglia and/or cortical regions of the central nervous system (CNS) is one pathological hallmark of PD, α-syn pathology and inclusions are also reported in the olfactory bulb, brainstem, and the enteric nervous system (ENS). Therefore, PD is not just a disease of the CNS but may involve the ENS and GI tract before overt neurodegeneration and movement symptoms. The pattern of α-syn pathology led Braak et al. to hypothesize that abnormal α-syn pathology may begin in the gut and propagate via a prion-like fashion into the CNS via the vagus nerve^6^. This “gut to brain” hypothesis is supported by α-syn + inclusion bodies in the ENS and vagus nerve in post-mortem tissues^6,7^. Further, the risk of developing PD was significantly reduced among individuals who received a full truncal vagotomy^8^. Experimental support for this hypothesis is observed in preclinical rodent models. Injections of α-syn pre-formed fibrils directly into the GI tract lead to pathology in the CNS in rodents^9–11^, also observed in similar experiments involving non-human primates^12^.

The majority of PD incidences are idiopathic and likely involve genetic and/or environmental interactions. The greatest genetic risk for idiopathic PD is having the eQTLs that upregulate expression of *SNCA*^13^, but their penetrance is very low, meaning they are not suffient to cause PD. It is possible that factors intrinsic to the GI tract, such as the gut microbiome, may contribute to the etiology or progression of PD pathogenesis, possibly in interaction with *SNCA (*PMID: 32566740). The gut microbiome consists of trillions of microbes that inhabit the GI tract and play important physiological roles across many aspects of health and disease. Numerous studies to date have highlighted compositional differences in the gut microbiome between persons with PD compared to neurologically healthy controls including the detection of altered abundances in several microbial species^14–16^. Meta-analyses of studies conducted using 16 S sequencing^16,17^, and more recent shotgun studies^14,18^ consistently show PD microbiome is associated with reduced levels of taxa and microbial pathways for degradation of plant-based fibers and short-chain fatty acid production, and increased levels of *Lactobacillus*, *Bifidobacteria*, *Klebsiella*, *Methanobrevibacter*, *Escherichia coli*, certain *Streptococcus* species, *Alistipes*, and opportunistic pathogens including *Porphyromonas* species. Experimental models have demonstrated varying levels of contribution by the gut microbiome to aspects of PD pathology including both α-syn dependent and toxicant-induced model systems^19–23^. Whether the gut microbiome is directly involved in the etiopathogenesis of PD in humans is unknown.

While experimental studies suggest the composition of the gut microbiome can impact α-syn-mediated disease pathogenesis, it remains unclear how PD-associated dysbiosis of the gut microbiome arises in an individual. Elucidating how the gut microbiome is first impacted in those with PD and subsequently shifts over disease progression is key for understanding how these experimental contributions ultimately relate to disease. One possibility is that underlying α-syn-mediated dysfunctions lead to an altered intestinal environment which impacts the gut microbiome composition. In the current study, we sought to test three major outcomes: (1) determine whether α-syn overexpression impacts gut microbiome composition in mice, (2) establish whether this dysbiosis is present from birth or develops with age, and (3) identify the specific microorganisms that likely drive this dysbiosis. We longitudinally sampled fecal pellets for 12 months from transgenic (TG) mice highly overexpressing α-syn under a Thy1 promoter (Thy1-SYN “line 61” TG mice)^24^ and their non-transgenic (NTG) littermate controls and performed shotgun metagenomics followed by taxonomic profiling. Our analysis revealed significant, age-dependent changes in the gut microbiome of TG mice compared to NTG control mice, indicating α-syn overexpression alone can drive dysbiosis in vivo. Differential abundance analysis of individual species revealed a substantial (greater than 10-fold) and highly significant (FDR < 0.02) reduction in the abundances of *Lactobacillus* and *Bifidobacterium* species in TG mice as compared to NTG mice. Certain species of *Lactobacillus* and *Bifidobacterium* in humans are consistently found highly enriched in PD^14,16^. The results from this study indicate α-syn overexpression in mice is sufficient to drives significant, age-dependent gut dysbiosis.

## Methods

### Mice

Female mice overexpressing human α-syn under the Thy1 promoter (Thy1-SYN “line 61” TG female mice) originally developed in the laboratory of Eliezer Masliah at the University of California San Diego^24^ MGI:5,435,401, were bred to BDF1 males (MGI:5649804) purchased from Charles River Laboratories (Wilmington, MA, USA) as previously described^25^. As the Thy1-SYN transgene insertion site is on the X chromosome, male α-syn transgenic mice exhibit age-dependent α-syn pathology, neuroinflammation including gliosis, and motor symptoms. In females, however, phenotype is inconsitent due to random X inactivation. We therefore exclusively used male mice in this study. Male and female mice were separated before genotyping 21 days post birth, and male TG and NTG genotyped littermates were co-housed (2–5 mice per cage) for the duration of the study. No animals were euthanized for the endpoints analyzed in this study. This study is reported in accordance with ARRIVE guidelines. All methods were performed in accordance with the relevant guidelines and regulations. The experimental methods were approved by the University of Alabama at Birmingham Institutional Animal Care and Use Committee.

### Fecal pellet collection and processing

#### Analytical sample

Fecal pellets were collected at four time points (1, 3, 6, and 12 months) from 22 TG and 32 NTG mice across 22 litters (total analytical sample *N* = 216). Nine out of the 22 litters included both TG and NTG mice and were included in sensitivity analyses (*N* = 11 TG and 22 NTG littermates). Fecal pellets were counted and collected from individual mice over 20 min in a novel clean cage environment by using sterile toothpicks, then immediately labeled and stored at -80℃ until DNA extraction.

### DNA isolation, library preparation, and next generation sequencing

Isolation of fecal DNA, sequence library preparation, and next generation sequencing were performed at CosmosID Inc. (Germantown, MD, USA). Isolation of DNA from fecal pellets was performed using QIAGEN DNeasy PowerSoil Pro Kit (Germantown, MD, USA) according to the manufacturer’s protocol, then quantified using Qubit™ 4 fluorometer and dsDNA HS Assay Kit (Thermofisher Scientific, Waltham, MA, USA). Isolated DNA was stored at -20 °C until library preparation. Sequence libraries were prepared using the Nextera XT DNA Library Preparation Kit (Illumina, San Diego, CA, USA) and Integrated DNA Technologies unique dual indexes (San Diego, CA, USA) with a total DNA input of 1ng. Genomic DNA was fragmented using a proportional amount of Illumina Nextera XT fragmentation enzyme. Unique dual indexes were added to each sample followed by 12 cycles of PCR to construct libraries. Sequence libraries were purified using AMPure magnetic beads (Beckman Coulter, Brea, CA, USA) and eluted in QIAGEN EB buffer. Sequence libraries were quantified using the Qubit™ 4 fluorometer and dsDNA HS Assay Kit. Libraries were then sequenced on an Illumina HiSeq X platform with 150 bp paired-end sequencing. Number of reads ranged from 1 M to 5 M per sample.

### Bioinformatic processing of sequences

We used FastQC (https://www.bioinformatics.babraham.ac.uk/projects/fastqc/*)* and MultiQC^26^ to check the quality of raw sequences, followed by processing of sequences using BBDuk v 38.92 (https://jgi.doe.gov/data-and-tools/software-tools/bbtools/*)* to remove adapters and PhiX sequences and quality trim and filter low-quality sequences. BBDuk was performed specifying `ftm = 5`, `tbo`, `tpe`, `qtrim = rl`, `trimq = 25`, and `minlen = 50` as described previously^14^. Next, all sequence reads mapping to the most recent version of the Mus musculus genome reference (GCA_000001635.9_GRCm39_genomic.fna) were removed using BBSplit v 38.92 with default parameters. Low-complexity sequences were filtered using BBDuk specifying `entropy = 0.01`. Number of reads per sample ranged from 0.3 M to 3.1 M after processing.

### Taxonomic profiling

Taxonomic profiling was performed for processed sequences using MetaPhlAn v 3.0.14 with default parameters^27^ and resulted in the detection of 48 species from 29 genera, ranging from 5 to 22 species per mouse. Relative abundances of taxa (used for differential abundance testing) were calculated using MetaPhlAn default settings. To calculate count data (used for beta- and alpha-diversity-based analyses), MetaPhlAn was run again adding the `--unknown-estimation` flag to generate relative abundances with unknown estimation (i.e., relative abundances that take the proportion of “unknown” microbes in a sample into account), then relative abundances were multiplied by the total sequence reads of the sample.

### Statistical analyses

For all statistical analyses and visualizations, R v 4.3.1 (https://www.r-project.org/*)* was used.

### Beta- and alpha-diversity

To measure differences in gut microbiome composition between samples (beta-diversity), we used Aitchison distances. Aitchison distances were calculated for each time point by taking the Euclidean distances of centered log-ratio (clr) transformed species counts. The clr transformation was performed using the following formula in R:

log(*x* + 1)-mean(log(*x* + 1)).

where *x* is a vector of all species counts in a sample. Aitchison distances were used as input to principal component analysis (PCA) and permutational analysis of variance (PERMANOVA)^28^. PCA was performed to observe the beta-diversity among samples by first generating principal components (PCs) using the `prcomp` function in R, then plotting PC1 and PC2 for each time point using the `autoplot` function from the ggfortify v 0.4.16 R package (https://cran.r-project.org/web/packages/ggfortify/index.html*).* PERMANOVA was performed to test for differences in beta-diversity between TG and NTG groups for each time point using the `adonis2` function from the vegan v 2.6.4 R package (https://cran.r-project.org/web/packages/vegan/index.html*)* specifying permuted P-values to be calculated with 9,999 permutations.

To measure intra-sample diversity of the gut microbiome (alpha-diversity), two diversity metrics were calculated (observed richness, and Shannon-Weiner index) using the `diversity` function in vegan. Differences in alpha diversity between TG and NTG groups at each time point were tested using linear regression (via the `lm` function in R) adjusted for the total sequence count per sample, and additionally, PC1 from PCA for 1, 3, and 6 months. Both total sequence count and PC1 were standardized using the `scale` function in R before testing.

### Differential abundance testing

We used two methods to test the difference in abundances of species and genera between TG and NTG groups: MaAsLin2 and ANCOM-BC. The tests included 32 species and 19 genera captured during the 12th month of sampling. MaAsLin2 was used to perform linear regression on log2-transformed relative abundances as implemented in the MaAsLin2 v 1.14.1 R package^29^. MaAsLin2 was run using default parameters with the following exceptions: `min_prevalence` was set to `0.05` making the effective sample size *N* = 3, `normalization` was set to `NONE` as we are already inputting relative abundances (which are normalized by the total sequence count per sample), and `standardize` was set to `FALSE` as standardization was performed for quantitative variables before running MaAsLin2. ANCOM-BC was used to calculate and perform testing on bias-corrected abundances (i.e., abundances corrected for uneven “sampling fractions” across samples) as implemented in the ANCOMBC v 2.6.0 R package^30^. ANCOM-BC was run using default parameters with the following exceptions: `prv_cut` was set to `0.05` and `p_adj_method` was set to `BH`. P-values from differential abundance testing were multiple testing corrected separately for species- and genus-level comparisons using the Benjamini-Hochberg false discovery rate (FDR) method^31^. Significance was defined as FDR < 0.05 by both MaAsLin2 and ANCOM-BC. Fold change in TG compared to NTG of the relative abundance (MaAsLin2) or bias-corrected abundance (ANCOM-BC) of a species or genus was calculated by taking the exponent of the beta coefficient from MaAsLin2 or ANCOM-BC using base 2 (MaAsLin2) or the natural base (ANCOM-BC). We chose to calculate fold changes in this way as the beta coefficient is (1) adjusted for the total sequence depth of a sample, giving us a more unbiased measure of fold change than just using raw relative, and (2) derived from log-transformed values, resulting in fold changes less influenced by outlying data points.

Additionally, testing was performed for earlier time points (1, 3, and 6 months) for species that were found differentially abundant between 12-month TG and NTG to observe their differential abundance as a function of age. All differential abundance analyses were adjusted for total sequence count per sample, and analyses for 1, 3, and 6-month time points were additionally adjusted for PC1.

### Sensitivity analysis

To ensure results were robust to any litter effects, as some litters contained only TG or NTG mice, all aforementioned analyses (beta-diversity, alpha-diversity, and differential abundance testing) were performed again including only litters that resulted in both TG and NTG litter mates (9 mouse litters out of 22). No difference in methodology was implemented save the fact that TG and NTG sample sizes were reduced by 50% (*N* = 22 to *N* = 11) and 38% (*N* = 32 to *N* = 20), respectively.

## Results

Human observations strongly demonstrate that persons with PD harbor a gut microbiome that is compositionally different from healthy controls. Experimental data suggest contributions by certain gut bacteria to PD-relevant pathologies in rodent models. However, what drives these alterations to the intestinal environment and selects for these compositions is unknown. We therefore sought to determine whether α-syn overexpression, a pathological attribute associated with PD, is sufficient to induce gut dysbiosis in mice. We collected fecal pellets from 54 male mice, including 22 Thy1-SYN “line 61” mice (TG) and 32 non-transgenic littermate controls (NTG), at four consecutive time points: 1 month, 3 months, 6 months, and 12 months post-birth. Littermates provide for a near identical maternally derived microbiome between genotypes. DNA was extracted and shotgun sequencing was performed, generating an average of 1 M to 5 M sequence reads per sample before sequence QC and 0.3 to 3.1 M after QC. All samples passed QC. We used standard protocols for bioinformatics (bioBakery suite), taxonomic profiling (MetaPhlAn), and statistics (MaAsLin2) to assess bacterial compositions.

### Visual inspection of microbiome similarities as mice age

**Fig. 1:**
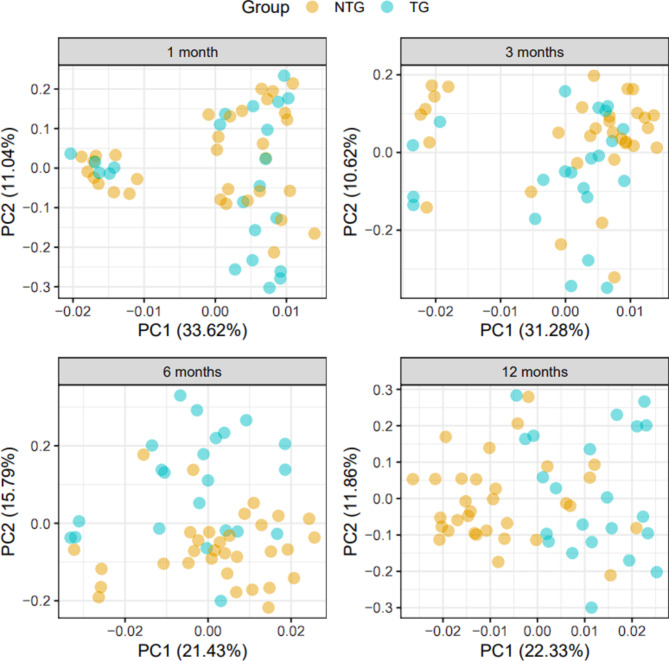
Inter-sample differences in gut microbiome composition (beta-diversity) of Thy1-SNCA transgenic and non-transgenic mice at 1, 3, 6, and 12 months. Principal component (PC) analysis was performed to visualize inter-sample differences in gut microbiome composition (beta-diversity) between Thy1-SNCA transgenic (TG, blue points, *N* = 22) and non-transgenic (NTG, orange points, *N* = 32) mice at each time point. Each point in the plots represents the gut microbiome composition of one unique mouse sample at a certain time point, and distances between points indicate the degree of similarity of a mouse sample to others. Percentages on the x- and y-axis correspond to the percent variation in gut microbiome compositions explained by PC1 and PC2. The differences between TG and NTG groups for each time point were formally tested using PERMANOVA (Table 1a).

We first extracted principal components (PC) from microbial abundance data and created PC distance plots for qualitative assessment of the similarity/dissimilarity of microbiomes at each age independently (Fig. 1). In mice aged 1 month, we detected an underlying structure (an unknown source of variation) which separated the samples into two clusters of 15 mice and 40 mice each. Of note, the smaller cluster consisted of mice from only 5 out of the 22 litters while the larger cluster consisted of mice from all 22 litters, suggesting clustering might be due to an anomaly that occurred in a small subset of litters. We ruled out genotype and time of sample collection, and were unable to pinpoint the cause of this clustering. This clustering incrementally decreased over time and vanished by month 12. The first principal component (PC1) explained 42% of the total variation seen in month 1. To control for this unexplained variation in early life, in downstream analysis, we adjusted for PC1 when testing ages 1, 3, or 6 months, but not 12 months because the clustreing had vanished with time. We do, however, note the appearance of a genotype effect that begins to define TG vs. NTG-derived microbiomes by 12 months of age (Fig. 1).

**Table 1:** Testing for differences in the global composition of gut microbiome (beta-diversity) between Thy1-SNCA transgenic and non-transgenic mice.

Age (months)	a. All mice (*N* = 54)			b. Litters with both TG and NTG (*N* = 31)		
	R2	F	*P*	R2	F	*P*
1	0.02	1.49	0.13	0.03	1.53	0.15
3	0.02	1.53	0.12	0.03	1.69	0.1
6	0.07	5.03	< 1E-4	0.05	2.19	0.02
12	0.1	6.01	< 1E-4	0.09	2.97	5E-4

Differences in the global composition of the gut microbiome between transgene (TG) and non-transgenic (NTG) mice were tested using permutational analysis of variance (PERMANOVA). All tests were adjusted for total sequence count per sample. Tests at 1, 3, and 6 months were also adjusted for the first PC from PCA (see Fig. 1). PERMANOVAs were performed once using all mice (a) and again using only mice from litters with both TG and NTG littermates (b). R2, the proportion of inter-sample variation in microbiome composition explained by differences between TG vs. NTG; F, pseudo-F statistic, the ratio between the amount of variance explained by differences between TG vs. NTG and the residual variance in the data; P, permuted P-value from PERMANOVA computed using 9,999 permutations.

### α-syn overexpression induces dysbiosis in the gut

We therefore conducted a formal statistical test to determine if the overall composition of the gut microbiome in TG mice was different from NTG mice (Supplementary Table 1). We tested the Atchison distances between samples (beta-diversity) using permutational multivariate analysis of variance analysis (PERMANOVA) to find whether NTG and TG-derived microbiomes were significantly different, and the estimated fraction of total variation explained by genotype (R2). At months 1 and 3 of age, there was no effect by genotype, neither qualitatively nor statistically (R2 < 2%, *P* > 0.1). However, by 6 months of age, the microbial beta diversity of the TG and NTG had differed significantly from each other (R2 = 7%, *P* = 0.0001) and this genotype effect continued to diverge such that by month 12, the difference between TG vs. NTG-derived microbiomes accounted for 10% of the total variance (R2 = 10%, *P* = 0.0001). Sensitivity analysis for litter-effect, using only 11 TG mice with their 22 NTG littermates, also showed no difference at 1 or 3 months, after which the genotypes begin to diverge progressively reaching significance at month 6 (R2 = 5%, *P* = 0.02) and 12 (R2 = 9%, *P* = 0.0009) (Table 1b). We therefore conclude that the TG genotype, defined by α-syn overexpression, leads to an age-dependent shift in microbiome composition that differs from their NTG littermates.

### α-syn overexpression results in reduced diversity in mouse gut microbiome

**Fig. 2:**
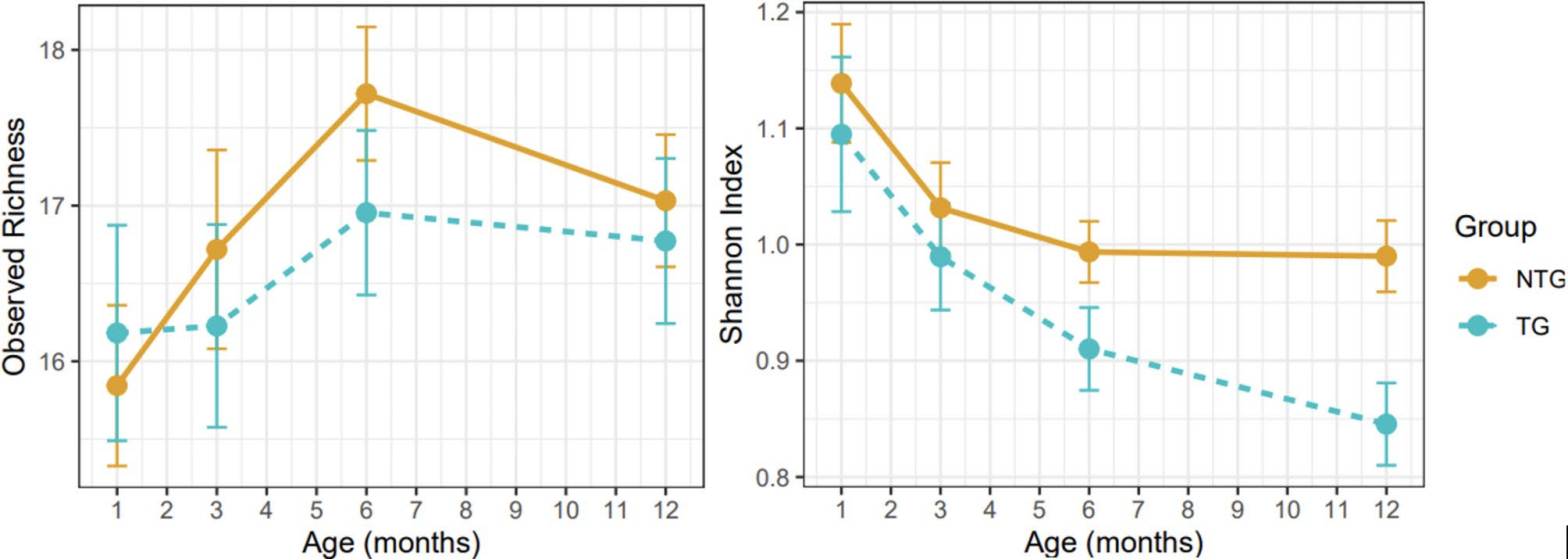
Thy1-SNCA transgenic mice exhibit a failure to attain diversity in gut microbiome composition compared to non-transgenic mice. Intra-individual diversity (alpha-diversity) was measured by observed richness, which reflects the number of species in each community, and by the Shannon index which accounts for the evenness of relative abundances as well as richness (lower Shannon index reflects lower diversity). In addition to this graphic presentation, differences in diversity metrics between transgene (TG) and non-transgenic (NTG) mice were formally tested using linear regression. All tests were adjusted for total sequence count per sample. Tests at 1, 3, and 6 months were also adjusted for the first PC from PCA (see Fig. 1). Tests were performed once using all mice, and again using only mice from litters with both TG and NTG littermates. The difference in Shannon diversity between TG and NTG becomes statistically significant at 12 months of age with *P* = 0.005 for all 54 mice and *P* = 0.06 for 31 mice in sensitivity analysis.

We next tested intra-individual variation (alpha-diversity) to determine whether the genotypes impacted the richness and evenness of the microbiome within an individual. Two methods were used to measure alpha-diversity: species richness (Fig. 2, left panel), which is simply the number of species present in a sample/community, and the Shannon index (Fig. 2, right panel), which takes into account both the number (richness) and relative abundance of species (evenness). As shown in Fig. 2 (left panel), initially after birth, both TG and NTG acquire more species with time, adding to the richness of the microbiome, reaching the peak at around age 6 months, after which they plateau or begin to lose the richness. While the trajectory over time is similar in NG and NTG, TG lags behind NTG at all time points and does not acquire the level of richness that NTG does at any point in the 12 months studied. As shown in Fig. 2 (right panel), TG had a lower Shannon index (less diversity) than NTG. While both TG and NTG exhibit a reduction in the Shannon index with aging, TG-associated microbiomes displayed a significantly steeper age-dependent loss of diversity than NTG (*P* = 0.005 for all mice at 12 months, *P* = 0.06 for the sensitivity analysis subset).

### Certain species of *Lactobacillus* and *Bifidobacterium* fail to bloom in α-syn overexpressing mice

Given our observations thus far, we have observed that TG and NTG mouse microbiomes diverge with age with the greatest difference occurring at 12 months of age. Our next step was to identify the specific bacterial species that drive the compositional shifts underlying this dysbiosis. We therefore designed a two-step analysis to specifically answer which taxa are driving the dysbiosis and when through the life of the mouse these changes appear. First, we conducted a microbiome-wide association study (MWAS) where we tested the differential abundance of species and genera at month 12, when the divergence was the greatest, and identified taxa that were significantly altered between genotypes. Then, we longitudinally traced the relative abundances of the specified TG-associated taxa throughout aging at 1, 3, 6, and 12 months of age.

A total of 48 species and 29 genera were captured in the whole dataset after stringent MetaPhlAn thresholds were applied. Of these, 32 species and 19 genera were represented within the 12-month-aged dataset. We conducted species-level and genus-level MWAS, at a 12-month time point, once with all 54 mice (setting significance at FDR < 0.05) and once with the 31 littermates (sensitivity analysis, requiring a trend as seen in 54 mice and calling it significant at *P* < 0.05). Species-level MWAS revealed 6 species that had significantly lower abundance in TG than in NTG (Table 2a). These included three species of *Lactobacillus*, each reduced by 14 to 50-fold in TG α-syn overexpressing mice as compared to NTG wildtype mice. Namely, *L. intestinalis* (fold change in TG vs. WT (FC) = 0.13, FDR = 1E-4), *L. reuteri* (FC = 0.07, FDR = 2E-3), and *L. johnsonii* (FC = 0.08, FDR = 2E-03) were substantially decreased. The three other species included *Bifidobacterium pseudolongum* which was reduced by 20-fold (FC = 0.05, FDR = 5E-4), *Muribaculum intestinale* with a 7-fold reduction (FC = 0.14, FDR = 2E-3), and *Muribaculaceae bacterium DSM_10372* which was reduced by 5-fold (FC = 0.20, FDR = 2E-3). All 6 of these species were further confirmed to be reduced in TG in sensitivity analysis of littermate (Table 2b). Therefore, we conclude that these taxa are sensitive to α-syn overexpression.

**Table 2:** Species and genera with significantly different abundances in Thy1-SNCA transgenic mice compared to non-transgenic mice.

Taxa	*N* TG	*N* NTG	MaAsLin2 results					ANCOM-BC results				
			Mean RA TG	Mean RA NTG	*P*	FDR	FC [95% CI]	Mean BC-OA TG	Mean BC-OA NTG	*P*	FDR	FC [95% CI]
a. All mice (*N* = 54)												
Species												
*Bifidobacterium pseudolongum*	3	21	0.1%	14.8%	3E-5	5E-4	0.05 [0.02; 0.19]	0.28	10.09	2E-9	4E-8	0.03 [0.01; 0.1]
*Lactobacillus intestinalis*	5	26	1.1%	5.0%	5E-6	1E-4	0.13 [0.06; 0.29]	0.5	14.68	2E-9	4E-8	0.04 [0.01; 0.11]
*Lactobacillus johnsonii*	13	29	2.5%	9.3%	4E-4	2E-3	0.08 [0.02; 0.29]	2.15	29.35	1E-5	8E-5	0.08 [0.03; 0.24]
*Lactobacillus reuteri*	13	30	6.2%	12.2%	3E-4	2E-3	0.07 [0.02; 0.27]	3.36	51.85	2E-5	1E-4	0.07 [0.02; 0.24]
*Muribaculaceae* bacterium DSM 103720	15	29	1.1%	2.5%	4E-4	2E-3	0.2 [0.09; 0.47]	1.79	11.1	2E-5	1E-4	0.16 [0.07; 0.37]
*Muribaculum intestinale*	16	31	1.8%	2.7%	3E-4	2E-3	0.14 [0.05; 0.38]	1.97	13.17	2E-6	2E-5	0.15 [0.07; 0.33]
Genera												
*Bifidobacterium*	3	21	0.1%	14.8%	3E-5	6E-4	0.05 [0.02; 0.19]	0.3	9.47	6E-9	1E-7	0.04 [0.01; 0.11]
Unclassified *Muribaculaceae*	15	29	1.1%	2.5%	4E-4	3E-3	0.2 [0.09; 0.47]	1.91	10.42	6E-5	4E-4	0.18 [0.08; 0.37]
*Muribaculum*	16	31	1.8%	2.7%	3E-4	3E-3	0.14 [0.05; 0.38]	2.1	12.35	5E-6	5E-5	0.18 [0.03; 0.68]
b. Litters with both TG and NTG (*N* = 31)												
Species												
*Bifidobacterium pseudolongum*	1	13	0.1%	10.5%	5E-3	0.08	0.07 [0.01; 0.4]	0.26	5.82	1E-5	2E-4	0.05 [0.01; 0.19]
*Lactobacillus intestinalis*	2	17	1.1%	5.2%	1E-3	0.04	0.24 [0.11; 0.52]	0.49	15.13	4E-6	1E-4	0.03 [0.01; 0.14]
*Lactobacillus johnsonii*	7	18	3.3%	8.7%	0.03	0.15	0.11 [0.02; 0.71]	3.06	22.71	0.02	0.1	0.14 [0.03; 0.69]
*Lactobacillus reuteri*	7	19	7.7%	12.3%	0.01	0.11	0.08 [0.01; 0.49]	5.05	49.96	0.01	0.08	0.11 [0.02; 0.59]
*Muribaculaceae* bacterium DSM 103720	7	18	1.4%	2.5%	0.02	0.14	0.23 [0.07; 0.76]	2.37	10.02	0.02	0.1	0.23 [0.07; 0.78]
*Muribaculum intestinale*	8	19	2.8%	2.8%	0.02	0.14	0.15 [0.03; 0.68]	2.3	11.51	9E-3	0.08	0.21 [0.07; 0.67]
Genera												
*Bifidobacterium*	1	13	0.1%	10.5%	5E-3	0.09	0.07 [0.01; 0.4]	0.07	1.74	5E-6	1E-4	0.04 [0.01; 0.17]
Unclassified *Muribaculaceae*	7	18	1.4%	2.5%	0.02	0.13	0.23 [0.07; 0.76]	0.67	2.99	0.01	0.08	0.22 [0.06; 0.72]
*Muribaculum*	8	19	2.8%	2.8%	0.02	0.13	0.15 [0.03; 0.68]	0.65	3.43	5E-3	0.04	0.2 [0.06; 0.61]

Species- and genus-level differential abundance analysis between transgenic (TG) and non-transgenic (NTG) mice was performed on mouse samples collected at 12 months using linear regression with log2-transformed relative abundances as implemented in MaAsLin2. Testing was performed again using a second method, ANCOM-BC, to ensure the robustness of signals. The analysis was adjusted for the total sequence count per sample. Tests were performed once using all mice (a) and again using only mice from litters with TG and NTG littermates (b). Species and genera whose relative abundance differed between TG and NTG groups at multiple testing corrected significance of < 0.05 in all mice for both statistical methods are shown. Full results are provided in Supplementary Tables 2–5. N, number of mice in which taxa were detected; Mean RA, mean relative abundance; FDR, false discovery rate q-value calculated via the Benjamini-Hochberg method; FC [95% CI], fold change and 95% confidence interval in TG compared to NTG of the relative abundance (MaAsLin2) or bias-corrected abundance (ANCOM-BC) of a species, calculated by taking exponent of Beta using base 2 (MaAsLin2) or the natural base (ANCOM-BC); P, raw P-value from testing with MaAsLin2 or ANCOM-BC.

As suspected from species-level MWAS, genus-level MWAS identified *Bifidobacterium*, *Muribaculum*, and an unclassified genus in the *Muribaculaceae* family as being reduced in the α-syn TG mice (Table 2). Surprisingly, despite the reduction of three species of *Lactobacillus*, there was no signal for the overall *Lactobacillus* genus. **Supplementary Tables 2–5** contain the full MWAS results. *Bifidobacterium*, *Muribaculum*, and the unclassified *Muribaculaceae* each had only one species from those genera detected in this dataset (**Supplementary Table 2**), hence their relative abundances and fold changes were similar at both genus and species levels. Conversely, the *Lactobacillus* genus contained four species: *L. intestinalis*, *L. reuteri*, *L. johnsonii*, and *L. murinus (***Supplementary Table 2)**. The former three were individually significantly reduced in TG mice at 12 months of age. However, these species were relatively rare within the TG population, adding up to a combined relative abundance of only 9% in TG mice vs. 26% in NTG. In contrast, *L. murinus* is not only common but it was also elevated in TG mice, albeit not significantly (mean relative abundance of 22% in WT and 42% in TG, FDR = 0.15). Thus, when the four species are pooled together at the genus level, these differential abundances mask the effects at the species level (**Supplementary Table 3)**. Hence, *Lactobacillus*, the only genus with multiple and the most significant signals at species level MWAS was missed at genus level MWAS.

**Fig. 3:**
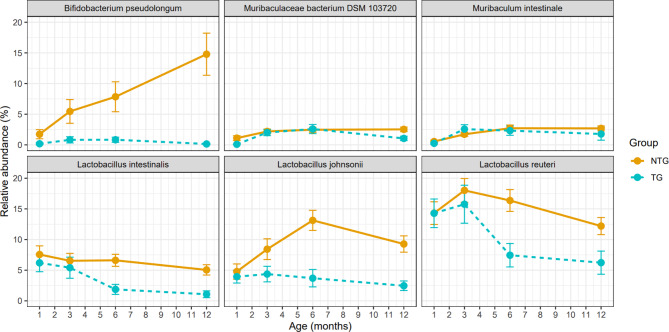
Changes over time in the relative abundance of species found differentially abundant between Thy1-SNCA transgenic and non-transgenic mice. Six species were found to be differentially abundant between 12-month-old Thy1-SNCA transgenic (TG, blue points and lines) and non-transgenic (NTG, orange points and lines) mice (Table 2), and their relative abundances were plotted over time to observe how they change with age. Each point represents the mean relative abundance of TG or NTG mice at a certain time point, and error bars extending from each point represent the standard error of the mean. Differences in relative abundances at each time point between TG and NTG mice were formally tested using MaAsLin2 (Supplementary Table 6).

The differential abundances of these specific species could be due to their enrichment in the NTG mice over time, their loss in the α-syn overexpressing TG mice, or simply differential initial abundances that were maintained throughout age. To differentiate between these possibilities, we tracked age-specific relative abundance profiles of the 6 species that characterized dysbiosis at 12 months of age (Fig. 3, **Supplementary Table 6)**. The most striking was *Bifidobacterium pseudolongum.* In NTG mice, *B. pseudolongum* had a mean relative abundance of 2% at month 1, 6% at month 3, 8% at month 6, and 15% at month 12. In TG mice, however, *B. pseudolongum* constituted only 0.2% of the microbiome at month 1, 0.8% in months 3 and 6, and 0.1% in month 12. Thus, TG mice were already depleted of *B. pseudolongum* by month 1, or it had never colonized effectively at birth. While NTG mice displayed a robust and progressive increase in the abundance of *B. pseudolongum* with age, this enrichment never occurred in the TG mice. Other TG-associated species do not show a significant difference at 1 or 3 months, maintaining their abundance at these early ages. The three *Lactobacillus* species diverge after month 3, and *Muribaculaceae and Muribaculum* species after month 6. Tracking the genera-level abundance by age would be identical to species-level tracking for all three TG-associated genera (*Bifidobacterium*, *Muribaculaceae*,* and Muribaculum)* because only one species was detected in these three genera levels. Overall, these data demonstrate that α-syn overexpression limits the growth of certain species within the gut microbiome and prevents a more diverse community from forming with age.

## Discussion

In this longitudinal observational study, we assessed the impact of α-syn overexpression on the gut bacterial community in mice. We identified that α-syn overexpression drives an age-dependent dysbiosis in mice, characterized by diminished diversity and a decrease in specific *Lactobacillus* and *Bifidobacteria* species.

In the present study, we selected the Thy1-hSNCA (line 61) α-syn overexpressing mouse model as it has been fairly well-characterized in its pathological progression. These mice produce 2-6-fold more synuclein protein across anatomical sites, leading to its accumulation^25^. In addition to α-syn pathological deposition in the CNS, mice display behavioral phenotypes, striatal dopamine depletion, and significant neuroinflammation in the absence of overt neurodegeneration^24,25,32^. While there are other mouse models available that display α-syn pathology in the CNS including various transgenic lines or injections of α-syn pre-formed fibrils^9^, these models are not as well characterized longitudenally. Line 61 mice express α-syn in the GI tract and also display characteristic slowed GI motility as early as 2.5-3 months of age^9,33,34^. This is notable, as we observe no genotype-dependent effect on the microbiome composition until after the 3-month time point. While not directly assessed, this suggests that α-syn overexpression induces GI deficits before the microbiome compositional shift in this model, as others observe GI dysfunctions arising concurrent and before this age^34^. It may be that a slowed intestinal environment selects against those taxa we observe increased with age in the NTG controls.

This α-syn overexpression model also begins to show mild to moderate increases in inflammatory markers beginning at 2 months of age and are subsequently exacerbated by 6 months of age^32^. Early changes include microgliosis, astrogliosis, and increased expression of the pro-inflammatory cytokine tumor necrosis factor (TNF) and the pattern recognition receptor Toll-like receptor 2 (TLR)^32^. Given a potential role for α-syn in immune activation, particularly via the interaction with TLR2 to mediate myeloid activation^35^, altered immune signaling in the GI tract may contribute alongside slowed motility to induce a microbiome compositional shift. This compositional shift may then feed back onto the immune system thus exacerbating inflammation in a pathological feed-forward cycle. In support, previous studies have shown elevated cytokine expression in the stool of individuals with PD^36^. One limitation of our longitudinal study herein was the inability to correlate inflammatory markers or α-syn overexpression with changes in the gut microbiome composition. Importantly, the compositional shift in the microbiome appears well before dopamine signaling is diminished in this model (~ 14 months of age)^25^ supporting the role of early changes in inflammatory pathways. Thus, the microbiome compositional shifts we observe may reflect a secondary pathology of α-syn overexpression. Future studies are warranted to dissect these mechanisms.

Two prior studies have conducted low-resolution 16 S profiling to explore the effects of transgenic α-syn production on the microbiome^16,22^. Despite differences in our sample sizes (5–10 vs. 54), genotypes (wild-type vs. α-syn), and sequencing method (16 S vs. shotgun metagenomics), we note a few key shared features. All studies demonstrate that α-syn overexpression leads to microbiome compositional changes that manifest shortly after birth and accelerate with age. The precise species that are selected for or against within each study may be secondary, driven by mouse strain, specific α-syn transgene, the microbiome facility, and other extrinsic factors. Each study also demonstrates that α-syn transgenic mice have a less diverse microbiome than wild-type controls. While Radisavljevic et al. attributes this to a loss of diversity, our data suggest instead it is an inability to gain diversity from a simpler newborn microbiome^37^.

It is noteworthy that *Lactobacillus* and *Bifidobacteria* emerge as the most significantly aberrant in both human PD and α-syn rodent models. *Lactobacillus* and *Bifidobactrium* species are generally enriched in PD^14,16,17^ however, mouse-associated species are not the same species we find altered in people with PD. Nonetheless, collective data suggest these taxa may be influenced by a-syn. One study has examined, and found evidence for, interaction of *SNCA* genotype with opportunistic pathogen levels in the PD gut microbiome^38^. Our present results call for investigating *SNCA* genotype with PD-associated *Lactobacillus* and *Bifidobacteria* species within human cohorts, which if true, would lend more credence to the relevance of this basic finding to the disease in humans. We posit that these taxa are sensitive to some features of the PD-associated intestinal environment that promotes or limits their abundances. These taxa may be sensitive to the alpha-synuclein induced environment of slowed intestinal motility and/or cross-feed from inflammation-mediated metabolites, and/or secreted monoamines, as examples. In vitro or defined in vivo systems to manipulate these aspects of the PD-associated intestinal environment would be needed to identify which may drive the abundance of these bacterial taxa. Given emerging data highlighting contributions of various bacterial species to promoting inflammation and synuclein pathology^21,23,39^, understanding why certain microorganisms come to be enriched in the PD-associated microbiome is essential.

There are many caveats in the model, and in the study, that limit how far one can extrapolate the results to PD. Setting aside the obvious biological differences between mice and humans, no existing rodent model fully recapitulates all attributes of PD. The Thy1 transgenic male mice exhibit age-dependent α-syn pathology, neuroinflammation including gliosis, and motor symptoms, but do not develop dopaminergic cell loss^25,40^. The transgene is on X chromosome, therefore, only males are used to avoid unpredictable phenotypes that result from X inactivation in females. The male-only limitation of the model prevents incorporating the sex effect that exists in PD or assessing sex differences. The transgene expresses very high levels of α-syn pan-neuronally starting before birth, a model which may be closer to having human *SNCA* gene multiplication than the low penetrance *SNCA* eQTLs that are associated with idiopathic PD. We chose this model because it is well characterized and recapitulates GI features; nonetheless, it would be of interest to study different models, with different insertion sites and consequently phenotypes. The study was designed to answer a basic key question: does *SNCA* transgene affect the gut microbiome, if so, is it age-dependent, and what taxa are affected most. Not knowing the outcome, we did not collect data for follow-up questions such as measuring inflammation and α-syn levels in gut and CNS. All limitations standing, results show a clear age-dependent relationship between *SNCA* genotype (greatest genetic risk factor for PD), which results in overproduction and therefore accumulation of α-syn protein (pathologic hallmark of PD), and a dysbiotic gut microbiome with reduced diversity (observed in every human PD study), and severely reduced levels of certain *Lactobacillus* and *Bifidobacterium* species (which are the genera most commonly associated with PD). These observations nonetheless pave the way for future studies, including verifying interaction of *SNCA* genotype with PD-associated *Lactobacillus* and *Bifidobacteria* in human cohortsand assessing direct and indirect influences of α-syn on both specific bacterial species and the microbiome as a whole, complete with measures of inflammation and α-syn levels in the gut and CNS.

## Conclusion

Overall, we found that α-syn overexpression in mice can drive alterations to the gut microbiome composition and limit the expansion of diversity with age. In addition, we identified specific bacterial species whose abundances change significantly in an age and genotype-dependent manner. Given emerging data on the potential contributions of the gut microbiome to PD pathologies, our data provide an experimental foundation to understand how the PD-associated microbiome may arise as a trigger or co-pathology to disease.

## Electronic supplementary material

Below is the link to the electronic supplementary material.

Supplementary Material 1

## Data Availability

The datasets generated and analyzed during the current study are available to the public with no restrictions at the NCBI Sequence Read Archive (SRA) under BioProject ID PRJNA1099939. Sequences on SRA are pre-QC. The post-QC taxonomic profiles generated here, along with sample metadata and analysis R code, are provided to the public with no restrictions at Zenodo [https://zenodo.org/record/10976403].
